# The gut microbiota of silkworm are altered by antibiotic exposure

**DOI:** 10.1002/2211-5463.13502

**Published:** 2022-11-14

**Authors:** Chengxu Li, Shuo Xu, Chunjie Xiang, Shixia Xu, Qihai Zhou, Junfeng Zhang

**Affiliations:** ^1^ Jiangsu Key Laboratory for Biodiversity and Biotechnology, College of Life Sciences Nanjing Normal University China; ^2^ Guangxi Key Laboratory of Rare and Endangered Animal Ecology Guangxi Normal University Guilin China; ^3^ School of Medicine & Holistic Integrative Medicine Nanjing University of Chinese Medical China

**Keywords:** antibiotic, bacteria, *Bombyx mori*, fungi, mulberry leaf, silkworm

## Abstract

In recent years, antibiotics have frequently been detected in soil, lakes, and rivers. Antibiotic residues in the environment may alter microbial structure and cause bacterial resistance, but their effect on interactions among host microbiota is still poorly understood. To investigate this issue, here we used silkworm (*Bombyx mori*) fed on antibiotic‐treated mulberry leaf as a model to explore the effects of antibiotic exposure on gut bacteria and fungi. We observed that elimination of fungi significantly reduced bacterial richness and diversity in silkworm intestine after exposure to the antifungal amphotericin B, while the elimination of bacteria dramatically increased the richness and diversity of fungi after exposure to the antibacterial ampicillin–streptomycin. Thus, antibiotic‐treated mulberry leaf significantly altered the community structure of microbiota in the gut of silkworm. Clearance of gut bacteria enhanced the correlation between gut fungi and leaf‐derived fungi, while clearance of gut fungi promoted abnormal proliferation of gut bacteria. These data provide a simple model to explore the comprehensive effect of diet‐derived bacteria, fungi, and antibiotics on gut microbiota.

## Graphical abstract

Here we investigated the effects of antibiotic exposure on intestinal microbiota interactions by feeding antibiotic‐treated mulberry leaves to silkworm. Our data suggest that antibiotics can change the structure and symbiosis of gut microbes, which provides a basis for further study of the underlying mechanisms by which antibiotics alter the microbial interactions in the intestine.

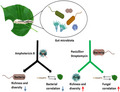

**Abbreviations**
LDA: linear discriminant analysis
LEfSe: linear discriminant analysis of the effect size
OTU: operational taxonomic unit
PCR: polymerase chain reaction

In recent years, antibiotics have been frequently found in the environment [1], such as soil [2], lakes, and rivers [3]. Antibiotic residues in the environment could alter the microbial structure [4] and cause bacterial resistance [5]. More worryingly, a review proposed that antibiotic residues in the environment could enter the gut through the food chain, and disturb the microbial balance, resulting in dysbacteriosis [6]. But until now, it is still unknown how antibiotics affect the interaction of bacteria and fungi in the intestine.

Microorganisms exist widely in nature, with high richness and diversity, and their interactions are conducive to realize a variety of functions, which plays an important role in maintaining the stability of the ecosystem [7]. Over the past decade, more and more research on microbial interaction attracted much attention, and the microbial cooperative phenotypes played a central role in many functions, including quorum sensing, biofilm formation, antibiotic resistance, and pathogenesis. Rakoff‐Nahoum et al. [8] tested the evolved cooperation within Bacteroidales and found that diet was the key factor affecting the bacterial cooperation during growth. Durán et al. [9] investigated the rhizosphere microbial community of *Arabidopsis thaliana* and detected mostly negative correlations between bacteria and filamentous fungi, especially bacterial microbiota was essential for plant survival and protection against root‐derived filamentous fungi. However, Xu et al. [10] found that the correlations between the core endophytic bacteria and fungi in *Oxytropis glacialis* were mainly synergistic. These inconsistent results might be explicated by different hosts. The factors affecting microbial interaction and their underlying mechanism remained poorly understood.

Intestinal microbiota are the largest and most complex microecosystem, affecting a variety of host activities, including digestion, immune response, and pathogen defense [11]. Host genetics, dietary habits, and environmental factors profoundly influence and shape the community structure of gut microbiome [12, 13]. A previous investigation showed that the dominant genera were *Streptococcus*, *Enterococcus*, and *Pseudomonas*, which could be distinctly altered by gender, age, state, or living environment [14]. Regarding the abiotic factors, temperature is the most crucial factor among environmental factors. Du et al. [15] found that high temperature (34 °C) obviously changed the gut microbiota of *Bombyx mori*, and the most noteworthy point was that the dominant microbiota changed from *Sphingomonas* and *Pseudomonas aeruginosa* to *Clostridium* and *Lactococcus*. In addition, *Methylobacterium* and *Buchner* were observed in the intestine of silkworms eating mulberry leaves, but not found in those fed with an artificial diet [16]. Comparison with silkworms eating mulberry leaves, the abundance of lipase bacteria was lower in the gut of silkworms eating tricuspid cudrania leaves [17]. Furthermore, bacteria and fungi are the main biological factors and active participants in the formation of the microecology. Their relationship includes commensalism, amensalism, mutualism, and competition [18]. However, it is still unclear whether altered bacteria or fungi exert an impact on the other organisms. Jones et al. [19] investigated the factors that influence gut microbiomes of fall armyworm (*Spodoptera frugiperda*) and corn earworm (*Helicoverpa zea*), and found that the host plant (diet) had a greater impact on gut communities than egg source (genetics). Thus, it can be speculated that diet is the key factor affecting the gut microbiota.

To overcome the great challenge of complex interactions among diet, bacteria, and fungi, here we designed a simple experiment on *B. mori*, a kind of lepidopteran insect feeding on mulberry leaves, to explore the association among diet, bacteria, and fungi based on exposure of antibiotics clearing the gut bacteria or fungi. The preliminary findings showed that the clearance of gut bacteria promoted the correlation between diet‐derived fungi and gut fungi, which provided a theoretical basis for advanced investigation on the underlying mechanisms of the interaction between gut bacteria and fungi.

## Materials and methods

### Sample collection

The silkworm species were “Suchao 2,” which were incubated in the laboratory at 37 °C in a dark incubator. According to the previous literature [20], the worms were divided into three groups: (a) the worms fed with fresh mulberry leaves were referred to as the control group; (b) the worms fed with fresh mulberry leaves treated by 10 μg·mL^−1^ amphotericin B solution (Sigma Chemical Co.St., Louis, MO, USA; CAS1397‐89‐3) were regard as the amphotericin group; (c) the worms fed with fresh mulberry leaves treated at 400 U·mL^−1^ penicillin and 400 μg·mL^−1^ streptomycin solution (TransGen Biotech Co.St., Beijing, China; FG1010‐01) were regard as the penicillin + streptomycin group. In addition, the antifungal amphotericin B could remove the gut fungi, and the antibacterial penicillin and streptomycin could delete the gut bacteria. In addition, the fresh mulberry leaves were regarded as the mulberry leaf group. After the worms were fed from hatching to the fifth instar larvae, the feces and leaves were sampled for microbial profiling.

### Total DNA extraction, PCR amplification, and high‐throughout sequencing

Total DNA was extracted from the mulberry leaves and feces of *B. mori*, and 1% agarose gel electrophoresis was used to control the quality of DNA. Using the extracted DNA as a template and amplified by polymerase chain reaction (PCR). The V3‐V4 region of the bacterial 16S rRNA gene was performed using forward primer 341F (5′‐CCTAYGGGRBGCASCAG‐3′) and reverse primer 806R (5′‐GGACTACNNGGGTATCTAAT‐3′); the ITS1‐ITS2 region was amplified using the forward primer ITS1F (5′‐CTTGGTCATTTAGAGGAAGTAA‐3′) and reverse primer ITS2R (5′‐GCTGCGTTCTTCATCGATGC‐3′) [21]. The PCR was performed in a 20 μL mixture containing 10 ng DNA extract, 0.8 μL primer (5 μmol·L^−1^), 0.4 μL FastPfu Polymerase, 4 μL 5× FastPfu Buffer, 2 μL dNTPs (2.5 mmol·L^−1^), replenished to 20 μL with ddH_2_O. All samples were amplified on an ABI GeneAmp 9700 (Applied Biosystems, New York, USA) using the following parameters: initial denaturation at 95 °C for 5 min, 27 cycles of denaturation at 95 °C for 30 s, annealing at 55 °C for 30 s, elongation at 72 °C for 45 s, and an extension at 72 °C for 10 min, then stored at 10 °C [22]. After we purified and quantified the products of amplification, high‐throughput sequencing was performed on the Illumina PE250 platform (Shanghai Lingen Biotechnology Co., Ltd, Shanghai, China).

### Statistical analysis

The raw data were optimized by removing the low‐quality sequences using the qiime software (version 1.17, http://qiime.org), and chimeric sequences were identified and deleted using the uchime algorithm. The refined sequences were clustered to the operational taxonomic units (OTUs) by 97% similarity using uparse (version 7.1, http://drive5.com/uparse/), and the OTUs were assigned according to the SILVA database with a 70% confidence threshold. Alpha and Beta diversity were analyzed based on the original OTUs abundance. After homogenization of OTUs, the relative abundance, community composition, linear discriminant analysis of the effect size (LEfSe), linear discriminant analysis (LDA), and Venn analysis were carried out. In addition, spss software (version 21, International Business Machines Corporation, Armonk, NY, USA) was used to conduct the nonparametric test and Spearman correlation analysis. Visualization of the microbial symbiotic was realized by cytoscape software (https://cytoscape.org/).

## Results

### Alpha diversity analysis

When the silkworms grew to the fifth instar larvae by feeding the antibiotics‐treated mulberry leaves, the fecal samples were collected. Amphotericin B‐exposure caused elimination of gut fungi. The PCR amplification and agarose electrophoresis detection results showed that the negative amplification rate of the 18S rRNA gene ITS1‐ITS2 region of silk worm feces was 85% (17/20), and six negative fecal samples were randomly selected as the amphotericin group for bacterial sequencing. Meanwhile, penicillin–streptomycin exposure caused the elimination of gut bacteria; the negative rate of the V3‐V4 region of 16S rRNA gene in silkworm feces was 90% (18/20), and six negative fecal samples were randomly selected as the penicillin + streptomycin group for fungal sequencing. At the same time, six mulberry leaves were collected as the mulberry leaf group, six fecal samples of silkworms fed the fresh mulberry leaves were selected as the control group, and these samples were conducted for both bacterial and fungal sequencing. Ultimately, the average of 37 830 ± 6702 bacterial sequences and 44 136 ± 9361 fungal sequences were obtained from each sample, and 607 bacterial OTUs and 533 fungal OTUs were obtained according to 97% similarity.

Alpha diversity analysis is presented in Table 1. For bacteria, compared with the mulberry leaf group, the richness index (Ace, Chao, Observed OTUs) and diversity index (Shannon, Simpson) of silkworm intestinal bacteria in the control group were significantly increased (*P* < 0.05), but the bacterial richness and diversity decreased in the amphotericin group, indicating that the loss of fungi decreased the bacterial richness and diversity in the intestine of silkworms. For fungi, compared with the mulberry leaf group, the richness and diversity of the gut fungi were signally decreased in the control group (*P* < 0.05), and no conspicuous alternation of the richness of gut fungi was observed (*P* < 0.05), but the fungal diversity index was remarkably increased in the penicillin + streptomycin group (*P* < 0.05), indicating that clearance of bacteria increases the diversity of fungi in the silkworm's intestine.

**Table 1:** Alpha diversity analysis of bacteria and fungi from the leaf to the gut of *Bombyx mori*. Compared with the control, the asterisk (*) means a significant difference (*P* < 0.05).

	Ace	Chao	Shannon	Simpson	Observed OTUs
Bacteria					
Control (*n* = 6)	178.2 ± 139.9	179.8 ± 139.5	2.533 ± 1.429	0.194 ± 0.101	168.2 ± 144.6
Amphotericin B (*n* = 6)	115.5 ± 10.9*	115.5 ± 12.4	1.845 ± 0.125*	0.256 ± 0.031*	105.4 ± 10.4*
Mulberry leaf (*n* = 6)	123.3 ± 18.8*	124.1 ± 21.0	1.740 ± 0.079*	0.277 ± 0.030*	105.4 ± 20.2*
*H* (*P*)	1.434 (0.488)	1.647 (0.439)	6.313 (0.043)	6.918 (0.031)	0.261 (0.878)
Fungi					
Control (*n* = 6)	95.5 ± 18.9	83.5 ± 30.3	2.598 ± 0.950	0.211 ± 0.262	75.0 ± 30.5
Ampicillin + Streptomycin (*n* = 6)	100.3 ± 57.8	97.5 ± 61.4	3.173 ± 0.629*	0.089 ± 0.050*	90.3 ± 56.3
Mulberry leaf (*n* = 6)	253.2 ± 53.6*	251.6 ± 55.1*	3.497 ± 0.254*	0.073 ± 0.031*	212.8 ± 39.9*
*H* (*P*)	9.018 (0.011)	9.029 (0.011)	3.71 (0.156)	2.946 (0.229)	8.404 (0.015)

### Clearance of gut bacteria promoted fungal similarity between gut and leaf

**Fig. 1:**
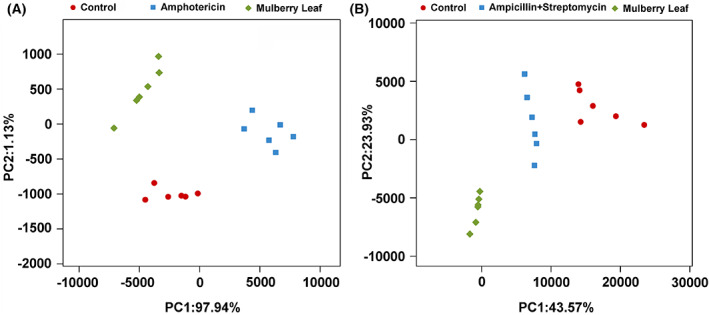
Effects of antibiotic treatment on intestinal microbiota of *Bombyx mori*. The gut bacteria were obviously altered after exposure to antifungal amphotericin B (A), and the gut fungi were also significantly altered after exposure to antibacterial ampicillin + streptomycin (B).

Principal component analysis showed that the bacterial and fungal community structures of the mulberry leaf were significantly different from that of silkworm gut. The bacterial or fungal community structure of the silkworm's intestine altered significantly, both in the amphotericin group and in the penicillin + streptomycin group (Fig. 1). In terms of bacteria, the distance between the control group and the mulberry leaf group was obviously detached, suggesting that the gut bacterial community differed distinctly from that of the mulberry leaf. And the bacterial community structure of the amphotericin group was obviously different from that of the control group, indicating that amphotericin B‐exposure induced clearance of gut fungi that altered the bacterial community structure (Fig. 1A). On the other hand, the fungal community was distinctly different between mulberry leaf and silkworm intestine, but the fungal community in the ampicillin + streptomycin group was more similar to the mulberry leaf group than that in the control group, indicating that ampicillin + streptomycin exposure induced clearance of gut bacteria increase the similarity of gut fungi and leaf (diet)‐derived fungi (Fig. 1B).

### Exposure of antibiotics distinctly altered the gut microbial composition

**Fig. 2:**
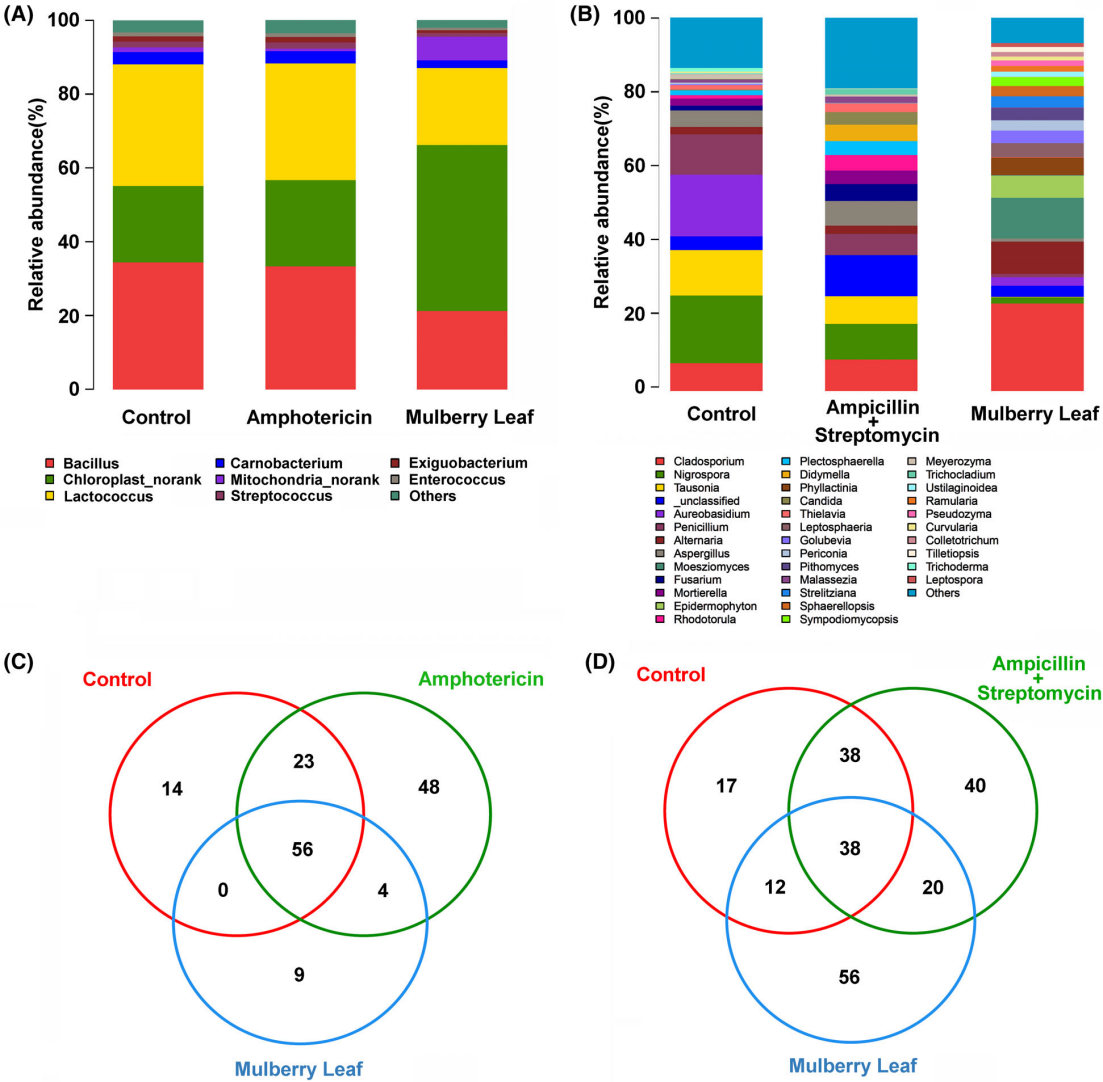
Genus‐level analysis of the microbial community structure. Here presented are the genus‐level structure of the bacteria treated by antifungal amphotericin (A), and genus‐level structure of the fungi treated by antibacterial ampicillin + streptomycin (B). The Venn diagram shows the shared bacterial genera (C) and fungal genera (D).

A total of 20 known bacterial phyla were detected, among which the relative abundances of three dominant bacterial phyla (*Firmicutes*, *Cyanobacteria*, and *Proteobacteria*) accounted for 90–96%. The relative abundances of four known fungi phyla (*Ascomycota*, *Basidiomycota*, *Chytridiomycota*, and *Mucoromycota*) ranked from 81% to 99%. At the genus level, eight dominant bacterial genera included *Bacillus*, *Chloroplast‐norank*, *Lactococcus*, *Carnobacterium*, *Mitochondria‐norank*, *Streptococcus*, *Exiguobacterium*, and *Enterococcus*. Based on the mulberry leaf group, the gut microbiota obviously altered but conserved some shared microbiota (Fig. 2). Firstly, here did not observe the apparent alteration between the amphotericin group and control group (Fig. 2A), suggesting that amphotericin B‐inducing clearance of gut fungi had little effect on the relative abundances of gut bacteria. The five dominant fungal genera including *Cladosporium*, *Nigrospora*, *Tausonia*, *Aureobasidium*, and *Penicillium*. On the other hand, the observed obvious fungal alteration between the penicillin+ streptomycin group and the control group (Fig. 2B), indicating that penicillin + streptomycin‐inducing clearance of gut bacteria exert deeply effect on gut fungi.

Among the 154 bacterial genera, Venn analysis results presented 56 shared bacterial genera (Fig. 2C), such as *Bacillus* (15.92%), *Chloroplast‐norank* (15.70%), *Carnobacterium* (15.53%), and *Lactococcus* (15.26%). Among the 221 fungal genera, the observed 38 shared fungal genera (Fig. 2D), included *Trichosporon*, *Trichomerium*, *Didymella*, and *Ceratocystis*.

**Fig. 3:**
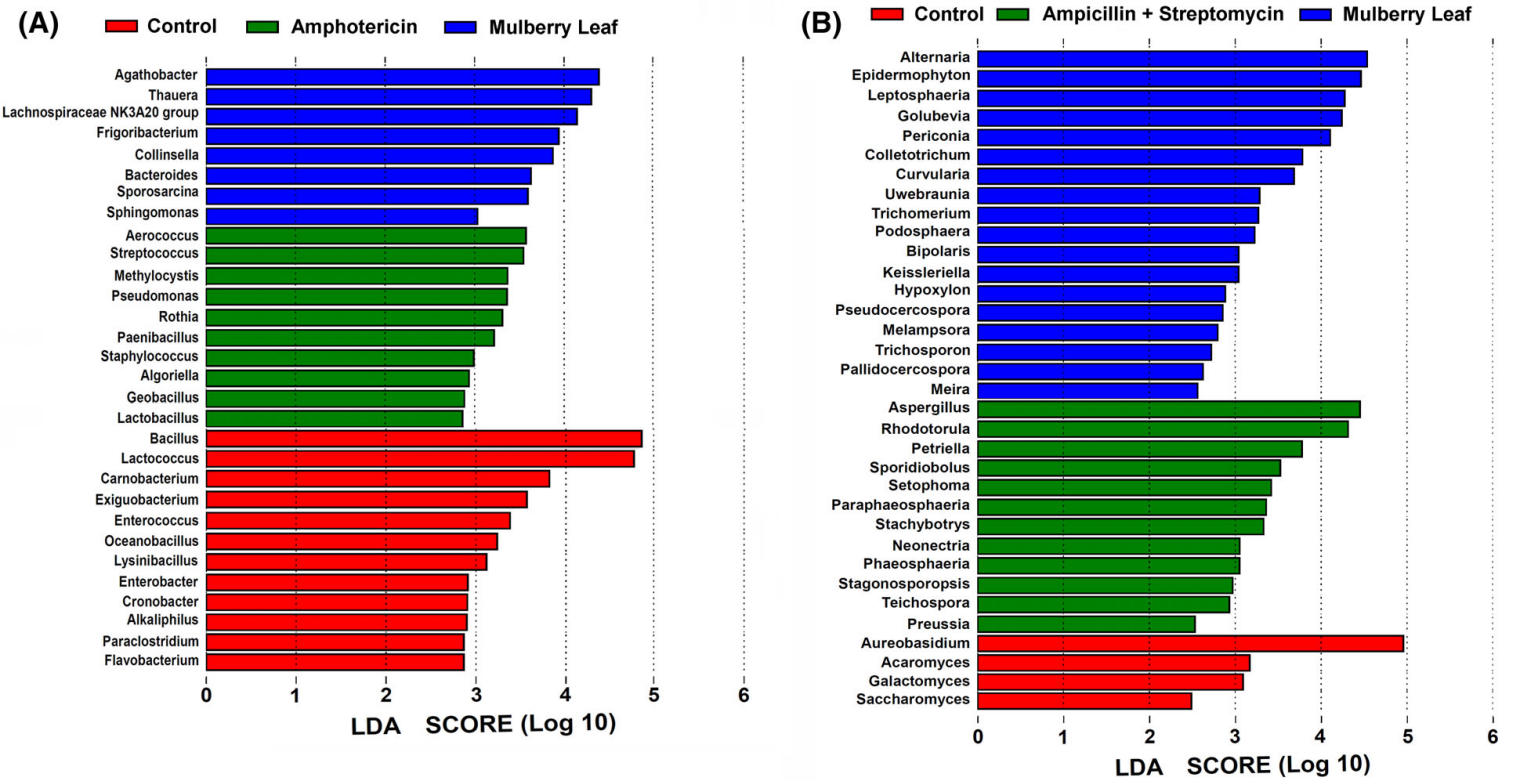
Enrichment of potential marker microbiota related to the antibiotic exposure. LDA resulted in 30 enriched bacterial genera (A) and 34 fungal genera (B) among the three groups.

Linear discriminant analysis was used to identify the potential microbial markers at the genus level (Fig. 3). Ten bacterial genera were enriched in the amphotericin group, such as *Aerococcus* and *Streptococcus*, belonging to the phylum *Firmicutes* (Fig. 3A). Twelve fungal genera were enriched in the penicillin+ streptomycin group, such as *Aspergillus*, belonging to the phylum *Ascomycota* (Fig. 3B). The results indicated that antibiotics exposure induced loss of the corresponding microorganism that deeply alter and shape the host's gut microbiota.

### Exposure of antibiotics deeply altered symbiotic relationships

**Fig. 4:**
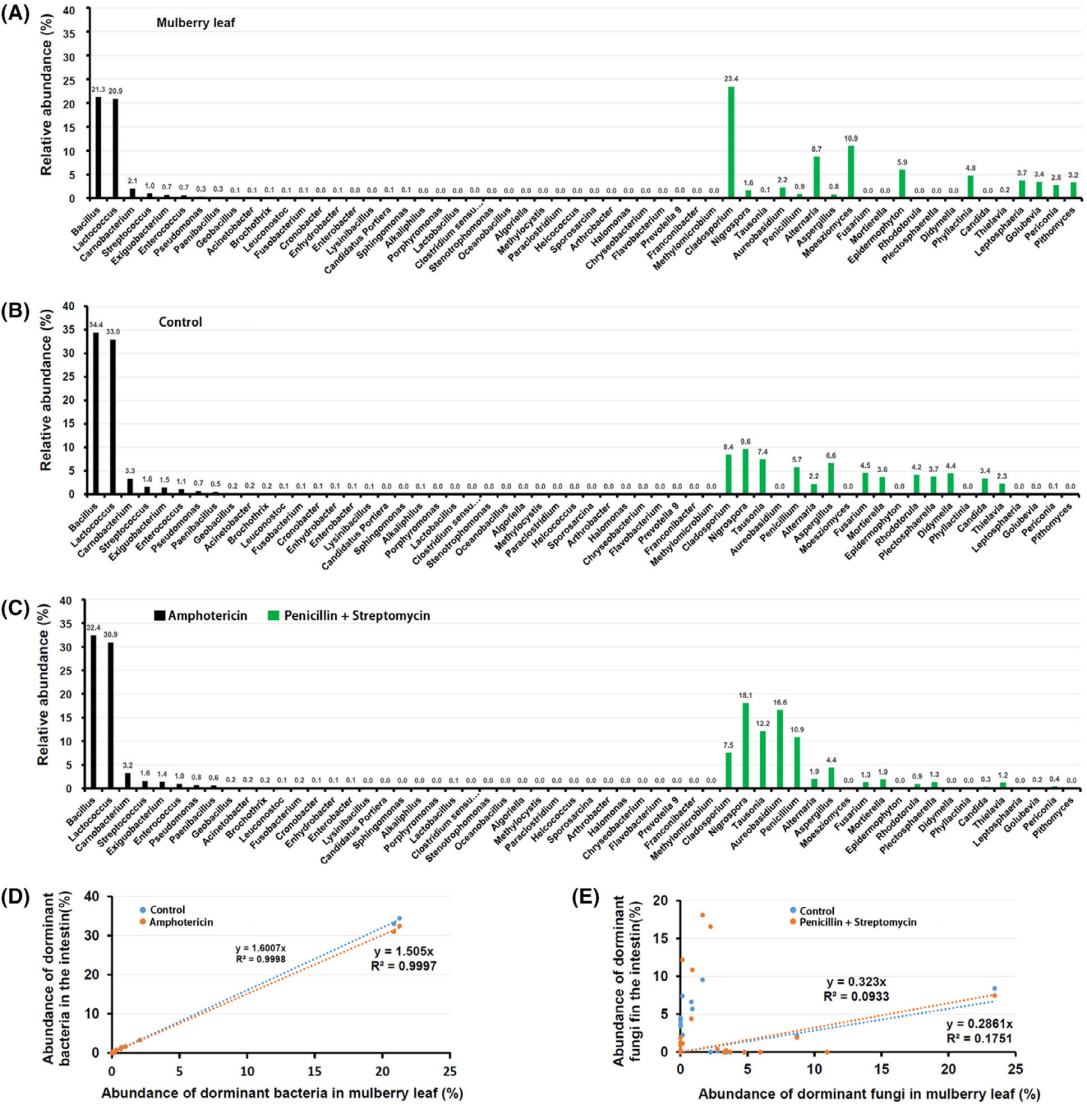
Exposure of antibiotics altered the tendency of microbial abundance. Thirty‐seven bacterial and 21 fungal genera are presented in the mulberry leaf (A), in the gut of control silkworm (B), and in the gut of silkworm exposed to antibiotics (C). Based on the microbiota of mulberry leaf, exposure of amphotericin B decreased the bacterial intercept (D), while exposure of ampicillin + streptomycin increased the fungal intercept (E).

Among the shared 56 bacterial genera in Fig. 2C and 38 fungal genera in Fig. 2D, bacteria and fungi genera with average relative abundance >0.1% were considered the core microbiota, so 37 bacterial genera and 21 fungal genera were selected to investigate the alteration of symbiotic relationships (Fig. 4). First, the bacterial abundance tendency in the mulberry leaf group (Fig. 4A) was homologous with that in the control group (Fig. 4B). Comparison to the control group, the bacterial abundance tendency did not obviously change in the amphotericin group; however, the tendency of fungal abundances obviously changed in the penicillin + streptomycin group (Fig. 4C). Spearman correlation analysis was conducted between the microbial abundances in the mulberry leaf and that in the gut. The nodal increment in the amphotericin group was lower than that in control group, suggesting that amphotericin B‐inducing clearance of gut fungi was weak in the bacterial correlation between the host intestine and the diet leaf (Fig. 4D). On the contrary, the nodal increment in the penicillin + streptomycin group was higher than that in the control group, suggesting that penicillin + streptomycin‐inducing clearance of gut bacteria enhances the fungal correlation between the gut and the diet leaf (Fig. 4E). The results suggested exposure of diet‐derived antibiotics exerted a different influence on the worm's gut microbiota.

**Fig. 5:**
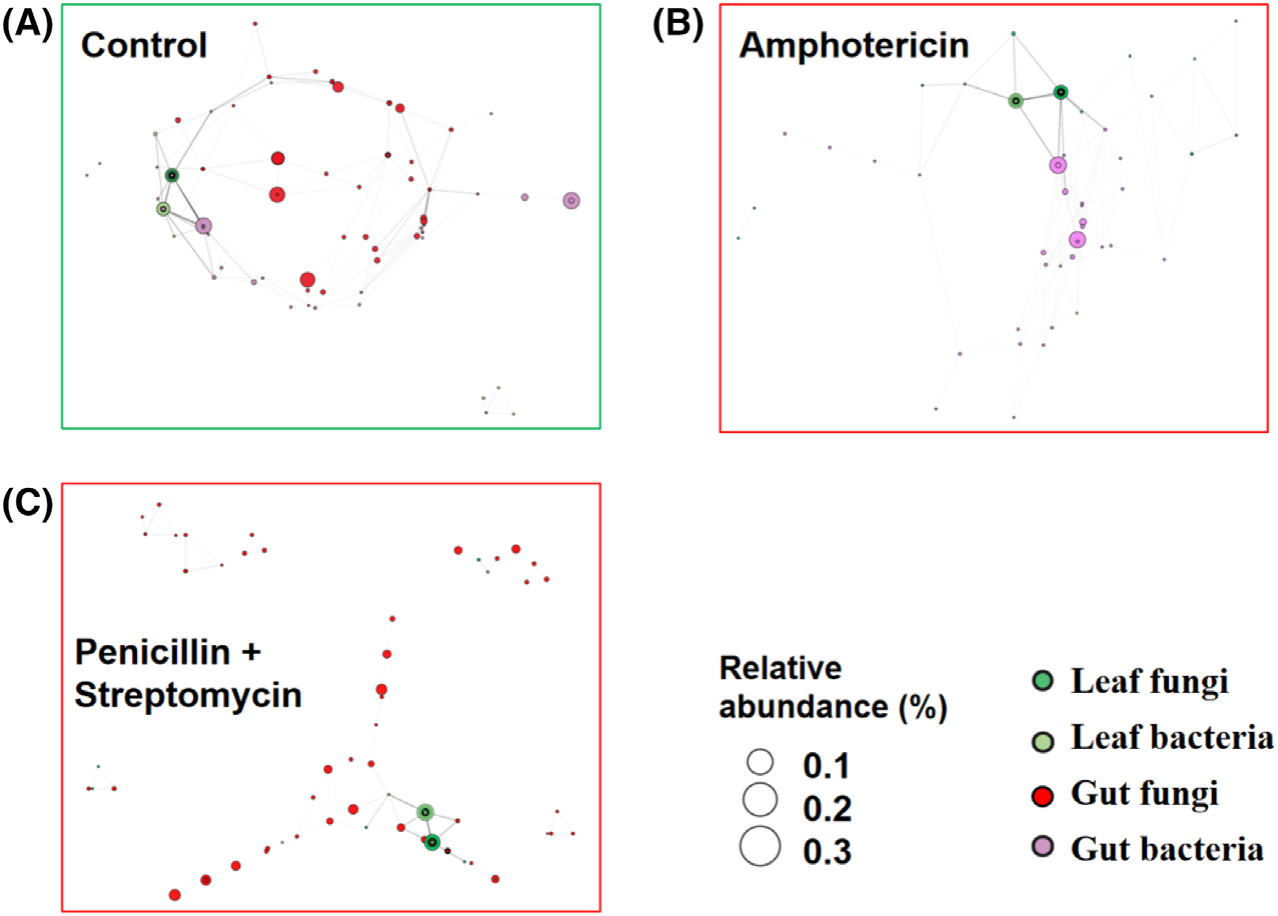
Exposure of antibiotics altered symbiotic correlations between the intestine and the mulberry leaf. Based on the dominant bacterial and fungal genera, the gut bacteria were closely related to the leaf‐derived bacteria in the control group (A). After exposure to the antifungal amphotericin B, the symbiotic network between gut bacteria and leaf‐derived bacteria was scattered, suggesting the bacterial relationship strength was weakened (B). After exposure to the antibacterial penicillin + streptomycin, the symbiotic network between gut fungi and leaf‐derived fungi became stronger, suggesting that the fungal relationship strength was enhanced (C). The size of the node means the relative abundance and the string length is negatively related to the correlation coefficient (*r*).

Finally, based on the 79 fungal genera and 37 bacterial genera, the symbiotic networks were constructed according to the latest literature [23] (Fig. 5). The dominant bacteria and fungi in the mulberry leaf group gathered more closely than that in the control group, suggesting that the intestinal environment obviously dispersed the strong correlation of leaf microbiota (Fig. 5A). In the amphotericin group, the symbiotic network was scattered between gut bacteria and leaf‐derived bacteria, suggesting amphotericin B‐inducing loss of gut fungi weakens the bacterial relationship between the intestine and the diet leaf (Fig. 5B). However, the symbiotic network between gut fungi and leave‐derived fungi became stronger in the penicillin + streptomycin group, suggesting that penicillin + streptomycin‐inducing loss of gut bacteria enhances the fungal relationship between the intestine and the diet leaf (Fig. 5C). These results indicated that exposure of antibiotics distinctly altered the commensal relationships in the worm's gut.

## Discussion

The pollution situation of antibiotics in the environment is now getting worse in the world, but little is known about the potential effects on the gut microbiota. As an extremely complex ecosystem, there is a continuous interaction among the microbiota in the human digestive tract [24]. In the foreseeable future, the interaction among the gut microbiota and host health will still be a hotspot [25]. Thus, the silkworm, with the simplest diet, was used as a model to overcome the complexity of diet‐driving intestinal microbial interaction in the mammals besides human. The present study used bacterial and fungal sequencing to explore the community structure and interaction of bacteria and fungi from the mulberry leaf to silkworm intestine. The preliminary results showed that the diet exposure of penicillin and streptomycin could cause the loss of gut bacteria, which had a significant impact on the fungi. And vice versa, the diet exposure of amphotericin B could cause the loss of gut fungi, which had a significant impact on the bacteria. Exposure of antibiotics could inhibit or kill the responding microbes, which would significantly alter the correlation between the silkworm's remaining microbiota in the digestive tract; especially the clearance of gut bacteria would promote the fungal correlation between the mulberry leaf and the intestine. In a word, antibiotics can change the diversity of microbial flora and destroy the balance of microecosystem in the gut of silkworm, thus affecting the development of sericulture. Obviously, it is necessary to standardize the use of antibiotics and develop new microecological preparations for silkworm to improve the health of the silkworm.

The hypothesis that the diet‐driving shape of gut microbiota has been gradually confirmed by mounting evidence [26, 27], and diet‐driving alteration of the gut microbiota has been suspected to be responsible for the increasing prevalence of chronic diseases such as obesity and inflammatory bowel disease [28]. For example, our epidemical investigations showed that the risk of gastric cancer was significantly related to the bacteria but not the fungi in the case of matching the lifestyle, the phenomena were observed both in the oral and fecal samples, which suggested that lifestyle played a key role in driving the alteration of gut microbiota, especially the fungi [21, 29]. An animal investigation also showed that the gut mycobiome of healthy mice was shaped by the environment and correlated with metabolic outcomes in response to diet [30]. Comparison to the diet leaf microbiota, the results of LDA showed that 12 bacterial genera and four fungal genera were markedly increased in the intestine of silkworms, two dominant bacterial genera (*Bacillus* and *Enterobacter*) were reported in the silkworm's intestine [31], and exposure of the antibiotics further promoted bacterial or fungal boost, such as *Aerococcus* and *Aspergillus*. The results were consistent with the explanation of antibiotics‐inducing dysbacteriosis.

Much evidence has found that bacterial and fungal mutualism play a major role in promoting and maintaining the host health and performance [32], suggesting that the symbiotic relationship between bacteria and fungi reflects the host's health and disease. Our data showed that the bacterial and fungal abundances in mulberry leaf were significantly different from that in silkworm's intestine. Interestingly, after exposure of antibiotics, clearance of gut fungi obviously enlarged the differences of bacterial structure between the intestine and the mulberry leaf; however, clearance of gut bacteria could shrink the differences of fungal structure between the intestine and the mulberry leaf and promote the crosslink between gut fungi and diet leaf‐derived fungi.

Since the industrial revolution, many kinds of antimicrobial agents are broadly used for bacteriostasis or sterilization as a natural or artificial synthetic chemical substance and play an essential role in medical treatment and significantly prolong the human life span [33]. However, the abuse of antimicrobial agents, especially the antibiotic, directly or indirectly causes a series of emerging human health problems [34]. Gudda et al. [35] conducted a risk assessment of antibiotic contamination of vegetables on the human intestinal microbiota, pointing out that the presence of dietary antibiotics may destroy some intestinal microbiota, disturb the balance of gut microbiota, and finally promote the abnormal proliferation of opportunistic and pathogenic bacteria. In our study, the clearance of gut bacteria induced by the diet exposure of penicillin + streptomycin promoted the correlation between gut fungi and diet‐derived fungi, while the clearance of gut fungi induced by diet exposure of amphotericin B promoted the abnormal proliferation of gut bacteria. The results provided new insight about the balance between gut and diet‐derived bacteria/fungi.

Up to now, there are still few studies on the effect of dietary antibiotics and microbiota on the gut microbiota this study used silkworm as a model to reveal the preliminary role of interaction among the bacteria, fungi, and diet exposed to antibiotics. The present data showed that exposure of dietary antibiotics significantly promoted the direct correlation between diet‐derived microbiota and gut microbiota, especially the fungi. Of course, the phenomena were observed in invertebrate, and a validated study is needed in vertebrates and to explore the underlying mechanism in health and disease. Anyway, the present data provide a simple model to present the underlying mechanisms of cross‐talking between diet‐derived microbiota, antibiotics, and gut microbiota, which will be an important clue for us to understand the potential hazards of antibiotic abuse.

## Conflict of interest

The authors declare no conflicts of interest.

## Author contributions

CL performed the data analysis and wrote the article. Shuo Xu and CX conducted the experiment and collected samples. Shixia Xu suggested the constructive comments of the study. QZ designed the study and performed the data analysis. JZ designed and supervised the study, and reviewed and revised the draft.

## Data Availability

The datasets presented in this study have been deposited in Entrez (https://www.ncbi.nlm.nih.gov/sra/PRJNA769808); the submission ID is SUB10504772.

## References

[1] Liu YH , Feng MJ , Wang B , Zhao X , Guo RX , Bu YQ , et al. Distribution and potential risk assessment of antibiotic pollution in the main drinking water sources of Nanjing, China. Environ Sci Pollut R. 2020;27:21429–41. 10.1007/s11356-020-08516-7 32274694

[2] Sun JT , Zeng QT , Tsang DCW , Zhu LZ , Li XD . Antibiotics in the agricultural soils from the Yangtze River Delta, China. Chemosphere. 2017;189:301–8. 10.1016/j.chemosphere.2017.09.040 28942256

[3] Sta Ana KM , Madriaga J , Espino MP . β‐Lactam antibiotics and antibiotic resistance in Asian lakes and rivers: an overview of contamination, sources and detection methods. Environ Pollut. 2021;275:116624. 10.1016/j.envpol.2021.116624 33571856

[4] Bacanlı M , Başaran N . Importance of antibiotic residues in animal food. Food Chem Toxicol. 2019;125:462–6. 10.1016/j.fct.2019.01.033 30710599

[5] Wu Y , Feng P , Li R , Chen X , Li X , Sumpradit T , et al. Progress in microbial remediation of antibiotic‐residue contaminated environment. Chin J Bioeng. 2019;35:2133–50 (In Chinese). 10.13345/j.cjb.190164 31814360

[6] Zhu H . Hazard and detection method of antibiotic residues in food of animal origin. Ind Technol Forum. 2016;15:64–5 (In Chinese). https://doi.org/CNKI:SUN:CYYT.0.2016‐14‐037

[7] Hao WQ , Zhang QG . Advances in microbial interaction research: from observation to prediction. J B Normal Univ (Nat Sci Ed). 2016;52:809–15. 10.16360/j.cnki.jbnuns.2016.06.019

[8] Rakoff‐Nahoum S , Foster KR , Comstock LE . The evolution of cooperation within the gut microbiota. Nature. 2016;533:255–9. 10.1038/nature17626 27111508 PMC4978124

[9] Durán P , Thiergart T , Garrido‐Oter R , Agler M , Kemen E , Schulze‐Lefert P , et al. Microbial interkingdom interactions in roots promote Arabidopsis survival. Cell. 2018;175:973–83. 10.1016/j.cell.2018.10.020 30388454 PMC6218654

[10] Xu GQ , Liu YX , Cao PX , Liu X . Definition and interaction network analysis of endophytic sclerotia microbiota from *Oxytropis glacialis* on the Qinghai‐Tibet Plateau. Microbiology. 2020;47:2746–58. 10.13344/j.microbiol.china.200307

[11] Ke P , Shao BZ , Xu ZQ , Chen XW , Liu C . Intestinal autophagy and its pharmacological control in inflammatory bowel disease. Front Immunol. 2017;7:695. 10.3389/FIMMU.2016.00695 28119697 PMC5220102

[12] Benson AK , Kelly SA , Legge R , Ma F , Low SJ , Kim J , et al. Individuality in gut microbiota composition is a complex poly‐genic trait shaped by multiple environmental and host genetic factors. Proc Natl Acad Sci U S A. 2010;107:18933–8. 10.1073/pnas.1007028107 20937875 PMC2973891

[13] Muegge BD , Kuczynski J , Knights D , Clemente JC , González A , Fontana L , et al. Diet drives convergence in gut microbiome functions across mammalian phylogeny and within humans. Science. 2011;332:970–4. 10.1126/science.1198719 21596990 PMC3303602

[14] Xu G , Sun ZL , Hu XL , Xue RY , Cao GL , Gong CL . Analyze the diversity of silkworm intestinal bacteria based on 16S rRNA gene sequence. Sci Seric. 2015;41:641–9 (In Chinese). 10.13441/j.cnki.cykx.2015.04.009

[15] Du BB , Liu YX , Wang HY , Yue L , Huang T , Xu SQ , et al. Effects of high temperature on intestinal microbial diversity of *Bombyx mori* . Chin Silkworm Assoc. 2016;186 (In Chinese).

[16] Hao CF , Li G , Sun X , Tang J , Qian HY , Zhao GD , et al. The diversity analysis of intestinal bacteria of silkworm larvae reared on different diets. J Insect Sci. 2019;62:61–72. 10.16380/j.kcxb.2019.01.007

[17] Feng W , Wang XQ , Zhou W , Liu GY , Wan YJ . Isolation and characterization of lipase‐producing bacteria in the intestine of the silkworm, *Bombyx mori*, reared on different forage. J Insect Sci. 2011;11:135. 10.1673/031.011.13501 22243438 PMC3391909

[18] Gao XF , Huynh BT , Guillemot D , Glaser P , Opatowski L . Inference of significant microbial interactions from longitudinal metagenomics data. Front Microbiol. 2018;9:2319. 10.3389/FMICB.2018.02319 30386306 PMC6198172

[19] Jones AG , Mason CJ , Felton GW , Hoover K . Host plant and population source drive diversity of microbial gut communities in two polyphagous insects. Sci Rep. 2019;9:17–34. 10.1038/s41598-019-39163-9 30808905 PMC6391413

[20] Li GN , Xia XJ , Parfait S , Zhao HH , Long YH , Zhu Y . Effects of fluoride on intestinal microflora of *Bombyx mori* . Acta Microbiol Sin. 2015;55:926–34. 10.13343/j.cnki.wsxb.20140450 26710611

[21] Xu S , Xiang CJ , Wu J , Teng YH , Wu ZF , Wang RP , et al. Tongue coating bacteria as a potential stable biomarker for gastric cancer independent of lifestyle. Dig Dis Sci. 2021;9:2964–80. 10.1007/S10620-020-06637-0 33044677

[22] Xu J , Xiang C , Zhang C , Xu BQ , Wu J , Wang RP , et al. Microbial biomarkers of common tongue coatings in patients with gastric cancer. Microb Pathog. 2019;127:97–105. 10.1016/j.micpath.2018.11.051 30508628

[23] Yang Y , Li G , Min K , Liu T , Li C , Xu J , et al. The potential role of fertilizer‐derived exogenous bacteria on soil bacterial community assemblage and network formation. Chemosphere. 2022;287(Pt 3):132338. 10.1016/j.chemosphere.2021.132338 34563774

[24] Hevia A , Delgado S , Sánchez B , Margolles A . Molecular players involved in the interaction between beneficial bacteria and the immune system. Front Microbiol. 2015;6:1285. 10.3389/FMICB.2015.01285 26635753 PMC4649051

[25] Erin RS , Jessica JM , Heidi MS . Conducting research on diet‐microbiome interactions: a review of current challenges, essential methodological principles, and recommendations for best practice in study design. J Hum Nutr Diet. 2021;34:631–44. 10.1111/JHN.12868 33639033

[26] Claesson MJ , Jeffery IB , Conde S , Power SE , O'Connor EM , Cusack S , et al. Gut microbiota composition correlates with diet and health in the elderly. Nature. 2012;488:178–84. 10.1038/nature11319 22797518

[27] Johnson AJ , Vangay P , Al‐Ghalith GA , Hillmann BM , Ward TL , Shields‐Cutler RR , et al. Daily sampling reveals personalized diet‐microbiome associations in humans. Cell Host Microbe. 2019;25:789–802. 10.1016/j.chom.2019.05.005 31194939

[28] David LA , Maurice CF , Carmody RN , Gootenberg DB , Button JE , Wolfe BE , et al. Diet rapidly and reproducibly alters the human gut microbiome. Nature. 2014;505:559–63. 10.1038/nature12820 24336217 PMC3957428

[29] Wu J , Zhang C , Xu S , Xiang CJ , Wang RP , Yang D , et al. Fecal microbiome alteration may be a potential marker for gastric cancer. Dis Markers. 2020;2020:3461315. 10.1155/2020/3461315 33014185 PMC7519184

[30] Mims TS , Abdallah QA , Stewart JD , Watts SP , White CT , Rousselle TV , et al. The gut mycobiome of healthy mice is shaped by the environment and correlates with metabolic outcomes in response to diet. Commun Biol. 2021;4:281. 10.1038/S42003-021-01820-Z 33674757 PMC7935979

[31] Yuan ZH , Lan XQ , Yang T , Xiao J , Zhou ZY . Investigation and analysis of intestinal bacterial population of *Bombyx mori* . Acta Microbiol Sin. 2006;46:285. 10.13343/j.cnki.wsxb.2006.02.024 16736593

[32] Guo Y , Narisawa K . Fungus‐bacterium symbionts promote plant health and performance. Microbes Environ. 2018;33:239–41. 10.1264/jsme2.ME3303rh 30270261 PMC6167116

[33] Spagnolo F , Trujillo M , Dennehy JJ . Why do antibiotics exist? MBio. 2021;12(6):e0196621. 10.1128/mBio.01966-21 34872345 PMC8649755

[34] Huang L , Yang CX , Liu SP . Hazard and detection method of antibiotic residues in food of animal origin. Guangzhou Chem. 2021;49:44–7. (In Chinese). 10.3969/j.issn.1001-9677.2021.05.016

[35] Gudda FO , Waigi MG , Odinga ES , Yang B , Carter L , Gao YZ . Antibiotic‐contaminated wastewater irrigated vegetables pose resistance selection risks to the gut microbiome. Environ Pollut. 2020;264:114752. 10.1016/j.envpol.2020.114752 32417582

